# Surgery beyond bodies: Soul surgery and social surgery

**DOI:** 10.3389/fsurg.2022.950172

**Published:** 2022-09-07

**Authors:** Bjørn Hofmann

**Affiliations:** ^1^ Centre of Medical Ethics, The University of Oslo, Oslo, Norway; ^2^ Institute of the Health Sciences, The Norwegian University of Science and Technology (NTNU), Gjøvik, Norway

**Keywords:** outcomes, mental, social, ethics, goal, aesthetics, gender

## Introduction

Surgery is impelled by triumphs and trailed by tragedies (1–5). While the triumphs are manifold, the tragedies have traditionally come in two kinds: harms and lack of benefits. Harms from surgery are well documented (6). So are treatments without benefit (7, 8). A recent review identified more than seventy general low-value surgical procedures where five high-volume, high-cost general surgical procedures alone could save the National Health Service more than €100 million yearly (9).

Lack of benefits and harms of surgery affect individuals, public health, and the standing of surgeons (10–12). However, a third type of challenge is pressing: *surgery beyond bodily benefit*, e.g., surgery with mental and social goals: cutting in bodies to improve self-esteem and social identity, i.e., “soul surgery” and “social surgery.”

Clearly, in surgery, as in medicine in general, there has been a move from targeting surrogate endpoints to hard endpoints, such as survival, morbidity, and functional status, and especially towards quality of life. Additionally, patient-reported outcome measures (PROMs) have received increased attention in surgery (13–17) as elsewhere. Hence surgery increasingly serves what matters to people.

The general move in the assessments of benefits of surgery from medical measures towards patient-centred measures calls for reflection, especially when the goals of surgery go beyond the body that is operated on. In particular, when the goals of surgery are mental (self-esteem) or social (recognition), it is crucial to reflect on the ethos of surgery. Moving the primary goals of surgery away from the site of incision may foster tragedies and hamper triumphs in the future. We therefore need to pay special attention to surgical goals that go beyond the body that is operated on, i.e., what can be called “soul surgery” and “social surgery.”

## Soul surgery

Soul surgery can be defined as surgery of the body to obtain primarily mental outcomes. Historically, there has been a strong belief in brain surgery to treat mental illnesses (“psychosurgery”). However, even when one believed that there was a clear connection between the site of incision and the mental effect, the outcomes were often poor or even devastating (18–20). More recently, surgery is used indirectly to influence people's mental health (21). No parts of the body are untouched by the scalpel to improve people's appearance and self-esteem. Female genital cosmetic surgery is but one example (22, 23).

There are several problems with “soul surgery.” First, it can be difficult to provide high-quality evidence for the mental outcomes of surgery (24–27). Second, sculpting bodies according to the current aesthetical ideals supports and spreads the norms (the “new normal”) that may cause the mental problems in the first place. Third, while surgery for self-esteem and other mental states may be good for individuals and employment of cosmetic surgeons, it may not be good for public health and social norms in general. Fourth, soul surgery may only target the symptoms and not the causes, diverting attention and resources from more effective approaches. Fifth, many types of soul surgery may cross the line between therapy and enhancement (28), e.g., when moving from reconstruction to construction. Sixth, it can be difficult to set the limits to soul surgery, e.g., when autonomous persons request to remove well-functioning organs or limbs (29). Last but not least, soul surgery can imply substantial harms, including harms well beyond the body.

In other words, when the outcome measures of surgery are remote to the operating site, benefits and harms must be assessed in a different (mental) realm, which demands special attention, competence, and care.

In addition to the intended mental effects of surgery, surgery also can have social goals – as they are closely interconnected.

## Social surgery

Corresponding to soul surgery, social surgery is defined as surgery of the body to obtain primarily social outcomes. Clearly surgery has implications beyond its biomedical outcomes. For example, a hip replacement or the reconstruction of a severely injured face influences the social life of the person. However, the challenges occur when incisions are made primarily to obtain social changes. Lobotomy was used to alter social behaviour and compulsory sterilization to prohibit reproduction. Genital modification (for religious, social, or cultural reasons) and surgery to confirm social constructs, such as gender identity, are other and more topical examples.

While this certainly illustrates the wide-reaching power of surgery and the intimate interconnection between the body, mind, and the social, it also challenges the traditional conception of surgery as a concrete physical biomedical discipline with traditional goals of curing, restoring biomedical function, and avoiding or reducing physical pain. Again, many of the same problems as with soul surgery emerge and demands competency and care – as well as delimiting definition of the discipline.

## Beyond bodily benefit

Clearly, the power of surgery reaches well beyond the bodies that are operated on. Bodily health affects mental wellbeing and social functioning. Curing cancer gives psychological and existential relief and makes it possible to function socially. Surgery therefore has beneficial mental and social (side) effects that should be fully appreciated. However, problems occur when mental and social effects become the primary outcome of cutting in bodies – and when the effects are remotely connected to the corporal intervention.

## Goals beyond the body

The trend of expanding the subject matter of surgery from the physical body to mental and social phenomena provides new opportunities to help people, but it also extends surgery's goals, outcome measures, and responsibility. This urges surgeons to familiarize with new phenomena, learn different aetiologies, acquire new competencies, decide on different endpoints, set new limits, and take on new duties and responsibilities. There is nothing wrong with changing or expanding ones goals, but it may warrant special care as it involves professional values, social norms, and alters the ethos of the profession (30).

Therefore, cutting in the bodies of mental and social beings certainly has mental and social effects – effects that we should be aware of and appreciate. However, mental norms and social values change over time. Their relationship with the bodies is indirect and contingent. What is considered to be a good mental or social effect today may not be so tomorrow. Self-esteem and social functioning depend on more than bodily appearance. Therefore, soul surgery and social surgery require skills of elusive norms and values. Table 1 provides an overview of some crucial issues, examples, and aspects to take into consideration when doing soul surgery and social surgery.

**Table 1:** Examples of issues and challenges to take into consideration when doing soul surgery and social surgery

Issues	Examples	Challenges to take into consideration
New phenomena	Mental states (self-esteem, self-conception) social functioning, social recognition, status, gender	*Conceptually*: Defining the concepts referring to the phenomena in meaningful ways to surgeons and others *Ontologically*: Clarifying that these phenomena are on par with the entities (tissues, organs) of surgery
Different aetiology	Non-mechanistic causality Involving aesthetical and social norms, perception, mental states, and social trends	*Epistemically*: Knowing that a certain bodily modification will change mental states, social status, and social relations. Knowing whether the obtained changes will be sustainable in altering moral and social settings (as aesthetical and social ideals change). *Practical*: Assessing mental and social (including normative) effects of surgery on individuals
New endpoints	Social functioning, self-esteem, social status	*Methodological*: Applying endpoints that are not traditional in surgery (“non-surgical endpoints”) and ascertain their relationship with surgery and traditional surgical outcome measures
Different moral goal	From pain, suffering, biological or physical dysfunction to wellbeing, mental, and social functioning, happiness	*Professionally*: ‐ Extending the scope and subject matter of surgery ‐ Extending responsibility ‐ Extending expectations and demands *Morally*: Broadening surgery’s moral goal *Practically*: To be updated and adjusted to changing aesthetical and social norms

Forming mental phenomena and social constructs by surgically shaping physical bodies disrupts traditional categories and transgresses basic boundaries. Moving the primary goals of surgery away from the site of incision, warrants critical reflection and is crucial to avoid tragedies and promote triumphs in the future of surgery.
